# Replacing process water and nitrogen sources with biogas slurry during cellulosic ethanol production

**DOI:** 10.1186/s13068-017-0921-y

**Published:** 2017-10-16

**Authors:** Yang You, Bo Wu, Yi-Wei Yang, Yan-Wei Wang, Song Liu, Qi-Li Zhu, Han Qin, Fu-Rong Tan, Zhi-Yong Ruan, Ke-Dong Ma, Li-Chun Dai, Min Zhang, Guo-Quan Hu, Ming-Xiong He

**Affiliations:** 1 Biomass Energy Technology Research Centre, Key Laboratory of Development and Application of Rural Renewable Energy (Ministry of Agriculture), Biogas Institute of Ministry of Agriculture, Section 4-13, Renmin Nanlu, Chengdu, 610041 People’s Republic of China; 2 Key Laboratory of Microbial Resources (Ministry of Agriculture, China), Institute of Agricultural Resources and Regional Planning, CAAS, Beijing, 100081 People’s Republic of China; 3 College of Environmental and Chemical Engineering, Dalian University, Dalian, 116622 People’s Republic of China

**Keywords:** Biogas slurry, Cellulosic ethanol, NaOH pretreatment, Enzymatic hydrolysis, *Zymomonas mobilis*

## Abstract

**Background:**

Environmental issues, such as the fossil energy crisis, have resulted in increased public attention to use bioethanol as an alternative renewable energy. For ethanol production, water and nutrient consumption has become increasingly important factors being considered by the bioethanol industry as reducing the consumption of these resources would decrease the overall cost of ethanol production. Biogas slurry contains not only large amounts of wastewater, but also the nutrients required for microbial growth, e.g., nitrogen, ammonia, phosphate, and potassium. Therefore, biogas slurry is an attractive potential resource for bioethanol production that could serve as an alternative to process water and nitrogen sources.

**Results:**

In this study, we propose a method that replaces the process water and nitrogen sources needed for cellulosic ethanol production by *Zymomonas mobilis* with biogas slurry. To test the efficacy of these methods, corn straw degradation following pretreatment with diluted NaOH and enzymatic hydrolysis in the absence of fresh water was evaluated. Then, ethanol fermentation using the ethanologenic bacterial strain *Z. mobilis* ZMT2 was conducted without supplementing with additional nitrogen sources. After pretreatment with 1.34% NaOH (w/v) diluted in 100% biogas slurry and continuous enzymatic hydrolysis for 144 h, 29.19 g/L glucose and 12.76 g/L xylose were generated from 30 g dry corn straw. The maximum ethanol concentration acquired was 13.75 g/L, which was a yield of 72.63% ethanol from the hydrolysate medium. Nearly 94.87% of the ammonia nitrogen was depleted and no nitrate nitrogen remained after ethanol fermentation. The use of biogas slurry as an alternative to process water and nitrogen sources may decrease the cost of cellulosic ethanol production by 10.0–20.0%. By combining pretreatment with NaOH diluted in biogas slurry, enzymatic hydrolysis, and ethanol fermentation, 56.3 kg of ethanol was produced by *Z. mobilis* ZMT-2 through fermentation of 1000 kg of dried corn straw.

**Conclusions:**

In this study, biogas slurry replaced process water and nitrogen sources during cellulosic ethanol production. The results suggest that biogas slurry is a potential alternative to water when pretreating corn straw and, thus, has important potential applications in cellulosic ethanol production from corn straw. This study not only provides a novel method for utilizing biogas slurry, but also demonstrates a means of reducing the overall cost of cellulosic ethanol.
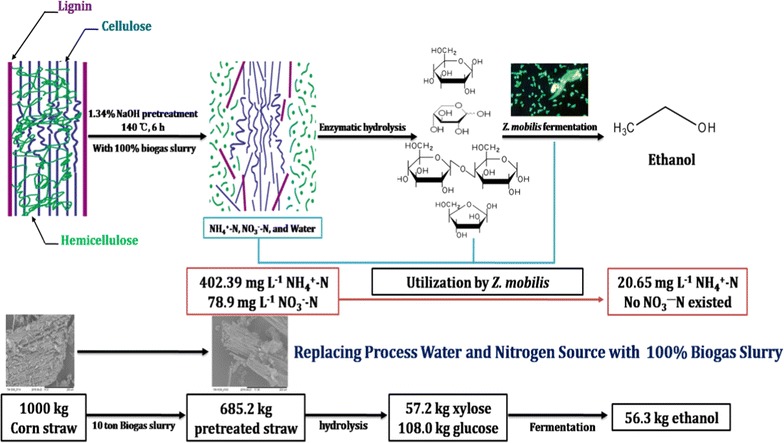

## Background

The production of biogas through anaerobic digestion (AD) is one of the most energy-efficient and environmentally friendly methods of generating bioenergy [1–3]. Recently, the production of biogas from biomass resources has gained more and more interest, especially in the European Union (EU) and China. According to the 2015 European Biogas Association (EBA) Biomethane & Biogas Report, there were 17,240 biogas plants and 8293 MWel of installed capacity in Europe by the end of 2014 [4]. The highest biogas producer in Europe at this time was Germany with 10,786 biogas plants that accounted for 62.56% of all biogas plants in the EU [4]. One of the earliest biogas consuming countries was China, which started use in 1929 as a means to treat agricultural waste and solve fuel shortages in rural areas [5, 6]. At the end of 2014, there were nearly 102,716 biogas plants treating agricultural waste in China, resulting in the production of about 2.01 billion m^3^ biogas per year [6].

Although AD has been used to treat different type of wastes and supply renewable energy worldwide, the majority of the nitrogen, ammonia, phosphate, and potassium in the agricultural wastes, particularly manure, remains in the biogas residue and slurry after AD. For example, up to 50% of the organic N is converted into ammonium (NH_4_–N) through AD, while the phosphate in the digestate remains unaffected [7]. Although the digestate is an improved fertilizer alternative in terms of mineral availability to crops [1], the low nutrient concentration in the digestate makes the cost of transportation and storage prohibitive. Importantly, the nutrient per-hectare and seasonal restriction of its application also prevent wide utilization, especially in Denmark, Germany, and France [7]. In recent years, the amount of biogas slurry produced has drastically increased as a result of the propagation of biogas in many Asian countries, especially China [8, 9]. The total nitrogen (N), phosphorus (P), and heavy metal content in the biogas slurries cannot be released directly into the environment [10]. In China, nearly 500 million tons of biogas digestate are produced annually, where only 60% is used as fertilizer or treated by aerobic process. Currently, the improper use and disposal of biogas slurry is a serious problem in China, resulting in environmental issues, such as eutrophication of surface water, over-the-limit levels of nitrate in the groundwater, and soil salinization [11, 12].

Due to the aforementioned issues, a number of researchers are focusing on utilizing biogas slurry in high-quality vegetable, carbohydrate, and biofuel production. For example, Liu et al. reported a method of soilless cultivation of high-quality vegetables using biogas manure [11]. In another study, Tan et al. showed that microalgae cultured with biogas slurry had an increased accumulation of intracellular carbohydrates [13]. Recently, the use of biogas slurry in the cultivation of microalgae for biofuel production has also received a great deal of interest [14]. As reviewed by Zhu et al. a scaled-up plan for simultaneous upgrading biogas slurry and digestate through microalgal cultivation has been proposed [14]. The previous studies on cultivating microalgal with biogas slurry have not only taken into account nutrient management, but also the accumulation of biomass for biofuel production. Although these studies provide potential means by which to utilize biogas slurry, large-scale methods of utilization are also necessary to counteract high yields of biogas slurry.

Currently, the ethanol industry has been successful in the United States of American (USA), Brazil, and China. In the bioethanol industry, water consumption has become an increasingly important consideration and a number of attempts have been made since the early 1990s to estimate water consumption during fuel production [15]. One study found the average amount of water consumed in the existing corn dry mill ethanol plants in USA from 1998 to 2007 ranged from 3.0 to 5.8 gallons water/gallon ethanol [15]. Due to technological improvements, the amount of water used has been nearly halved since 1998 and only 2.7 gals of water are required per gal ethanol produced from corn [16]. However, more water is required during cellulosic ethanol production at nearly 2.7–16.5 gallons water required per gallon ethanol produced [17]. Reducing the water used would decrease the overall cost of ethanol production. Another important factor in the bioethanol industry to consider is the provision of nutrients required for microbial cell growth, which can significantly increase the cost of large-scale production [18, 19]. For example, industrial nitrogen sources used for cellulosic ethanol production include corn steep liquor (CSL) and diammonium phosphate (DAP), which have been estimated to incur costs between $1.7 and 2.2 million/year per 200 million liters ethanol plant [6, 16]. Because biogas slurry contains both large amounts of wastewater and nutrients required for microbial cell growth, e.g., nitrogen, ammonia, phosphate, and potassium, it is a potential resource with which to replace process water and nitrogen sources during bioethanol production. Therefore, in this study, we proposed a method of cellulosic ethanol production that replaces process water and nitrogen sources with biogas slurry. Pretreatment with NaOH diluted in biogas slurry and enzymatic hydrolysis were used to degrade corn straw without adding fresh water, and ethanol fermentation was performed using ethanologenic *Zymomonas mobilis* without supplementing with additional nitrogen sources. Our study provides a novel method of producing cellulosic ethanol that uses biogas slurry and potentially reduces the cost of cellulosic ethanol production.

## Results and discussion

### Characteristics of corn straw after pretreatment with different concentrations of biogas slurry

The characteristics of untreated or treated corn straw are summarized in Table 1. Lignocellulosics, which contains hemicellulose, cellulose, and lignin, accounted for 52.78% of the dry matter of corn straw. After being pretreated with 1.34% NaOH (w/v) diluted in 100% of biogas slurry, the cellulose fraction was found to increase by 14.62%, from 23.18 to 26.57%. However, when fresh water was used, the cellulose fraction increases by only 11.66%. On the other hand, we also observed a significant decrease in lignin fraction in the two pretreated groups: 18.39% for biogas slurry group and 17.59% for fresh water group.

**Table 1:** Characteristics of corn straw

	Original corn straw	Corn straw pretreated with 100% biogas slurry	Corn straw pretreated with fresh water
NDF, % dry matter	52.78 ± 1.06	52.93 ± 0.51	51.84 ± 0.86
ADF, % dry matter	44.33 ± 1.26	43.83 ± 0.43	43.67 ± 1.14
ADL, % dry matter	21.15 ± 0.57	17.26 ± 0.53	17.43 ± 0.56
Cellulose (= ADF − ADL) % dry matter	23.18 ± 1.96	26.57 ± 0.35	26.24 ± 1.71
Hemicellulose (= NDF − ADF) % dry matter	8.45 ± 0.86	9.10 ± 0.89	8.17 ± 0.58
Lignin (= ADL) % dry matter	21.15 ± 0.57	17.26 ± 0.53	17.43 ± 0.56
N, % dry matter	1.08 ± 0.25	1.18 ± 0.72	1.24 ± 0.34
C, % dry matter	44.96 ± 2.34	34.38 ± 0.52	31.48 ± 1.56

All data are the average of triplicates with standard deviations of the means (*n* = 3) at *α* = 0.05

Raw corn straw was pretreated with 1.34% NaOH (w/v) diluted in concentrations of biogas slurry ranging from 0 to 100% (Table 2) for 6 h at 140 °C. After 1.34% NaOH (w/v) pretreatment in the absence of biogas slurry, the cellulose fraction increased by 13.2%, going from 23.18 to 26.24%, while the lignin and hemicellulose fractions slightly decreased, indicating that NaOH pretreatment reduces the crystallinity of corn straw.

**Table 2:** Concentrations of biogas slurry used in this study

Concentration of biogas slurry (%)	Biogas slurry (mL)	Fresh water (mL)	NaOH (g)
0	0	50	0.67
10	5	45	0.67
20	10	40	0.67
30	15	35	0.67
40	20	30	0.67
50	25	25	0.67
60	30	20	0.67
70	35	15	0.67
80	40	10	0.67
90	45	5	0.67
100	50	0	0.67

After pretreatment with 1.34% NaOH (w/v) diluted in 10–80% biogas slurry, the cellulose content increased from 23.18 to 30.44%, the hemicellulose content increased from 8.45 to 9.81%, and the lignin content stayed at 16 ± 1%. However, when the concentration of the biogas slurry was increased to 90 and 100%, the cellulose content decreased to 28.09 and 26.57%, respectively (Table 3). The loss of cellulose and hemicellulose likely followed pretreatment with NaOH diluted in fresh water and/or biogas slurry. This result indicates that the NaOH pretreatment efficiently disrupted the straw structure and also reducing its crystallinity.

**Table 3:** Characteristics of corn straw following pretreatment with different concentrations of biogas slurry

Biogas slurry (%)	Cellulose (% dry matter)	Hemicellulose (% dry matter)	Lignin (% dry matter)
0	26.24	8.17	17.43
10	12.66	4.31	15.22
20	12.03	3.78	15.81
30	15.47	4.89	15.29
40	23.44	9.02	18.43
50	24.24	9.68	17.65
60	27.55	9.53	18.81
70	29.76	9.27	16.54
*80*	*30.44*	*9.81*	*15.03*
90	28.09	9.47	16.15
100	26.57	9.10	17.26

The best results showed as italics

The effects of different concentration of biogas slurry on the yield of reducing sugars were also determined. As shown in Table 4, the maximum total yield of reducing sugars was 3.97 g/L (1.50 g/L glucose, 1.79 g/L xylose, and 0.68 g/L arabinose, respectively), which was achieved when a 50% biogas slurry was used during pretreatment. The lowest total yield of reducing sugars was 1.47 g/L (0.50 g/L glucose, 0.54 g/L xylose, and 0.43 g/L arabinose, respectively), which occurred when 70% biogas slurry used.

**Table 4:** Concentrations of reducing sugar after pretreatment with different concentrations of biogas slurry

Biogas slurry (%)	Glucose (g/L)	Xylose (g/L)	Arabinose (g/L)	Total reducing sugar (g/L)
0	0.74	0.82	0.38	1.94
10	0.61	0.71	0.52	1.84
20	1.08	1.20	0.81	3.08
30	0.57	0.67	0.47	1.71
40	0.70	0.67	0.61	1.98
*50*	*1.50*	*1.79*	*0.68*	*3.97*
60	1.39	1.68	0.64	3.71
70	0.50	0.54	0.43	1.47
80	0.46	0.48	0.58	1.51
90	1.33	1.57	0.63	3.53
100	0.83	1.35	0.60	2.78

The best results showed as italics

Degradation of cellulosic biomass is required during glucose production; however, the polymers in hemicellulose and lignin are difficult to hydrolyze into xylose, arabinose, and galactose. In lignified plant cells, cellulose and hemicellulose lignin are entrenched in the cells. Therefore, pretreatment with biogas slurry reduces the alkalinity and helps lift the lignin out of the cells, as well as performs part of the hemicellulose degradation needed to generate reducing sugars. The higher the concentration of reducing sugars after pretreatment, the better the removal of lignin and the degradation of cellulose and hemicellulose into fermentable sugar. Therefore, pretreatment of corn straw aids not only the removal of lignin, but also maximizes the retention of cellulose and hemicellulose. After pretreatment with 60% biogas slurry, the cellulose and hemicellulose contents were 27.55 and 9.53%, respectively (Table 3), which neared the maximum amount of cellulose and hemicellulose generated in this study, and the concentration of reducing sugars generated was 3.71 g/L (Table 4). Based on these results, the use of 60% biogas slurry during pretreatment may be optimal for the treatment of corn straw, as it increases the yield of reducing sugars, cellulose, and hemicellulose compared to the use of pure water. This indicates that biogas slurry can replace water for pretreatment of corn straw.

### Tabletop scanning electron microscopy analysis

**Fig. 1:**
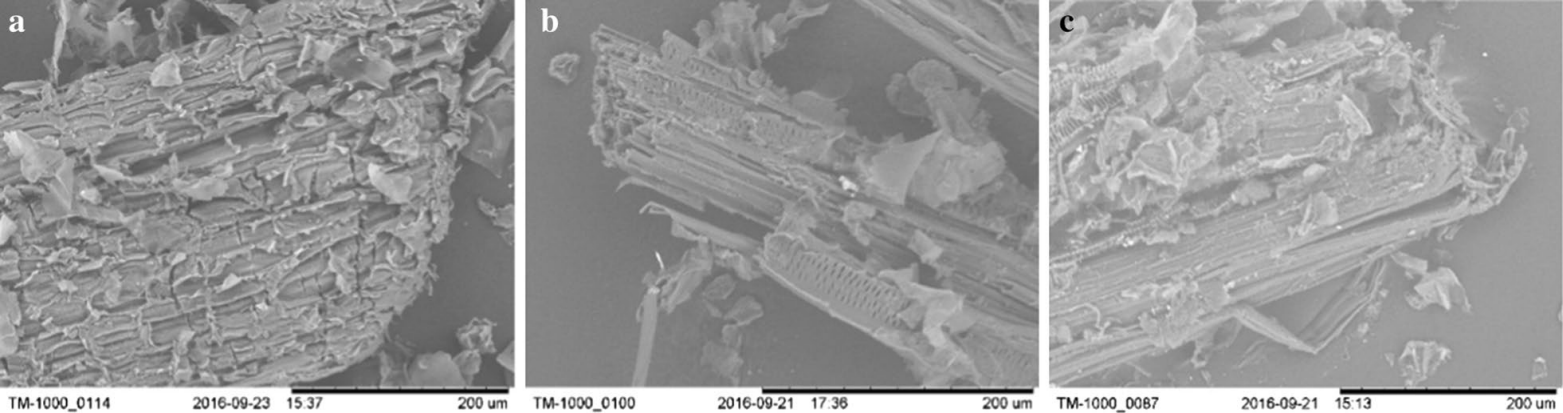
Visualization of corn straw by tabletop microscopy (× 500). **a** Untreated corn straw, and **b** corn straw pretreated with biogas slurry, and **c** corn straw pretreated with fresh water for 6 h at 140 °C with 1.34% NaOH (w/v) and 100 g L^−1^ substrate concentration

The structural changes of untreated corn straw and NaOH pretreated straw were analyzed by scanning electron microscopy (TM-1000). As shown in Fig. 1, the fibers of the pretreated corn straw appeared looser than the untreated straw with some holes in the fiber and thinner fiber stripes. This suggests that alkaline pretreatment efficiently breaks down the crystalline structure of the fiber, degrades hemicelluloses, and removes lignin to expose cellulose [20], which collectively enhance the hydrolysis of cellulase and, thus, help to improve the sugar yield.

### Enzymatic hydrolysis of pretreated corn straw with biogas slurry

After pretreatment with 1.34% NaOH (w/v) diluted in different concentrations of biogas slurry, enzymatic hydrolysis was performed. The hydrolysate contained cellobiose, glucose, and xylose. Cellobiose is a product of cellulose that has not been fully hydrolyzed. Cellulose is hydrolyzed to produce two molecules of glucose, which results in a lower concentration of cellobiose. A small amount of cellobiose in the hydrolysate shows that the length of hydrolysis was not sufficient and the cellulose was not hydrolyzed by the cellulase. As shown in Table 5, when the cellulose and hemicellulose contents increased in corn straw from pretreatment, the maximum concentrations of glucose and xylose generated were 2.77 and 1.22 g/L, respectively, when 40% biogas slurry was used.

**Table 5:** Concentrations of reducing sugar after enzymatic hydrolysis of corn straw pretreated with biogas slurry

Biogas slurry (%)	Glucose (g/L)	Xylose (g/L)	Cellobiose (g/L)	Total reducing sugar (g/L)
0	2.01	1.09	0.25	3.71
10	2.09	1.10	0.24	3.76
20	2.19	1.11	0.22	4.00
30	2.18	1.10	0.20	3.89
*40*	*2.77*	*1.22*	*0.23*	*4.62*
50	2.76	1.19	0.24	4.46
60	2.76	1.19	0.23	4.48
70	2.50	1.15	0.21	4.27
80	2.41	1.12	0.18	4.08
90	2.22	1.00	0.20	3.71
100	2.18	0.99	0.20	3.78

The best results showed as italics

### Continuous enzymatic hydrolysis of pretreated corn straw with biogas slurry

**Fig. 2:**
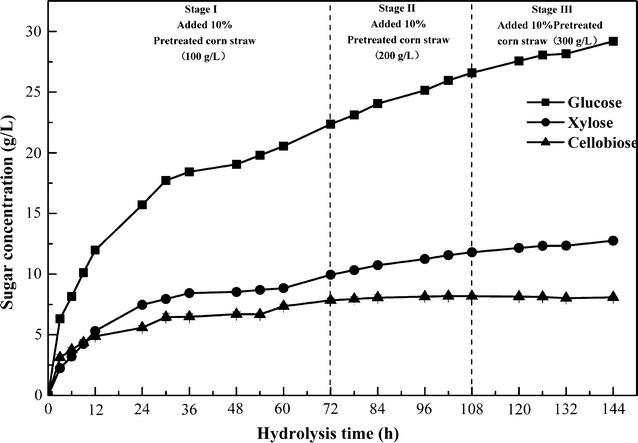
Changes in reducing sugars, glucose, and xylose during continuous enzymolysis using 100% biogas slurry treated. All data are the average of triplicates

Pretreatment and enzymatic hydrolysis using different concentrations of biogas slurry were performed. The largest amounts of cellulose and hemicellulose were generated when 80% biogas slurry was used (Table 3, showed as italics). After pretreatment with 1.34% NaOH (w/v), the highest total concentration of reducing sugars created was 3.97 g/L, which occurred when 50% biogas slurry was used (Table 4, showed as italics). After enzymatic hydrolysis of pretreated corn straw with biogas slurry, the highest total concentration of reducing sugars was 4.62 g/L from using 50% biogas slurry (Table 5, showed as italics). While the use of 100% biogas slurry did not maximize the cellulose content and total reducing sugar yield, this concentration of biogas slurry should be considered for the complete replacement of water. Therefore, 1.34% NaOH (w/v) diluted in 100% biogas slurry was used for continuous enzymatic hydrolysis. As shown in Fig. 2, the glucose and xylose concentrations reached 29.19 and 12.76 g/L, respectively, after continuous enzymatic hydrolysis of pretreated corn straw with 100% biogas slurry for 144 h. We observed the same trends in sugar yield in another fresh water group (data not shown), which supports the idea that biogas slurry can replace fresh water in corn straw hydrolysis. The concentrations of glucose and xylose increased rapidly over 36 h, and the concentration of reducing sugars in the hydrolysate increased as the length of enzymatic hydrolysis increased. At this stage, cellulase was completely in the substrate active center, and the enzymatic efficiency increased as the substrate concentration increased from 0 to 300 g/L (As shown in Fig. 2, Stage I, Stage II, and Stage III). However, after enzymatic hydrolysis for 72 h, the straw substrate likely reached its maximum concentration for cellulase, and the hydrolyzed reducing sugar reached a certain degree of inhibition of cellulase, resulting in reduced enzymatic efficiency and yield of reducing sugars within the next 72 h. However, the inhibition was not strong, and the concentrations of glucose and xylose in the hydrolysate increased as the length of enzymolysis increased. Cellobiose content remained stable after 72 h of enzymatic hydrolysis, indicating that β-glucosidase hydrolysis of cellobiose in cellulose was inhibited, because it was no longer able to hydrolyse cellobiose to glucose.

### Ethanol fermentation analysis

Ethanol fermentation by *Z. mobilis* ZMT2 was conducted using hydrolysate as the substrate and RM medium employed as a control. *Z. mobilis* ZMT2 has a unique Entner–Doudoroff (ED) metabolic pathway that uses anaerobic fermentation of hexoses, such as glucose, fructose, and sucrose, but not pentose, to produce ethanol.

**Fig. 3:**
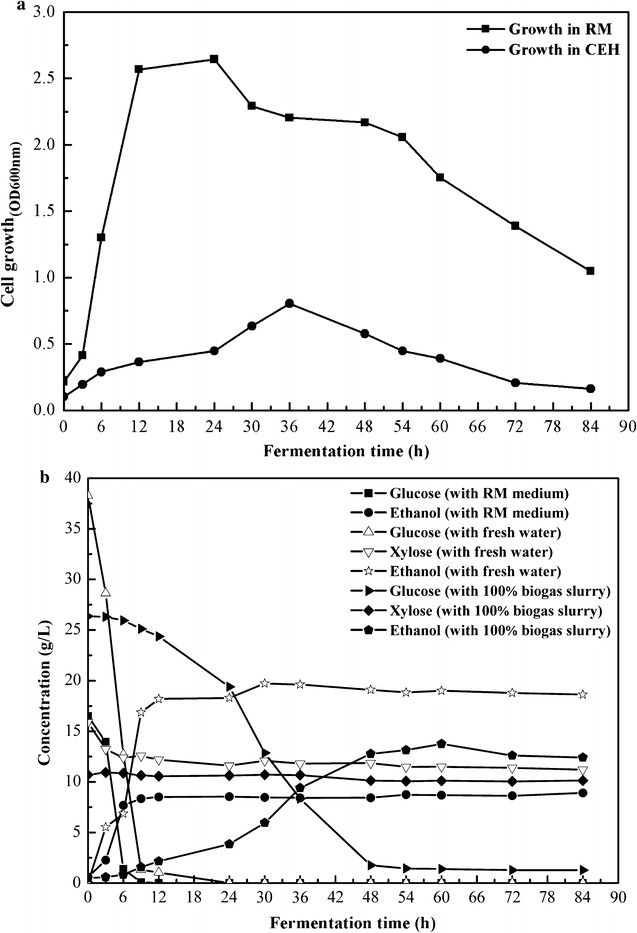
Bio-ethanol fermentation by *Z. mobilis* ZMT2 using continuous enzymatic hydrolysate of corn straw that had been pretreated with biogas slurry. **a** Growth curve of ZMT2. **b** CEH (Continuous Enzymatic Hydrolysate fermentation in the presence and absence of biogas slurry) and RM (control). All data are the average of triplicates

First, cell growth was compared between hydrolysate from biogas slurry pretreated corn straw and RM medium. As shown in Fig. 3a, the growth and cell density of ZMT-2 in the RM medium was significantly better than in the continuous enzyme hydrolysate. However, the cell density of ZMT-2 reached its maximum after culturing for 36 h in hydrolysate from biogas slurry pretreated corn straw. This indicates that the components of the hydrolysate from the biogas slurry pretreated corn straw were more complex than RM. In addition, the growth of ZMT-2 may be affected by some unknown environmental factors.

Second, sugar consumption was measured during ethanol fermentation. As shown in Fig. 3b, 20 g/L glucose was completely consumed and yielded 8.51 g/L ethanol within 12 h in RM medium, whereas only 8.6% of the total glucose was consumed yielding 2.15 g/L ethanol after 12 h in the 100% biogas slurry. However, when cultured for 60 h, 94.7% of the total glucose was consumed and 13.75 g/L ethanol was produced with a 72.63% ethanol yield from corn straw hydrolysate when 100% biogas slurry was used. All the glucose was completely consumed, and 19.71 g/L ethanol was generated after 30 h when corn straw hydrolysate without any biogas slurry was used, which was an ethanol yield of 71.22%. The slower rate of glucose conversion in the hydrolysate medium may be due to some unidentified inhibitors in the biogas slurry or produced during NaOH pretreatment, which remains to be examined in future studies.

Third, although biogas could be used for replacing nitrogen sources and process water during cellulosic ethanol production, the components of biogas slurry are easily susceptible to season, biogas fermentation process, fermentation material, etc. This may affect its utilization in ethanol fermentation due to altered inhibitor or ammonia nitrogen content.

In addition, because of the limitations of the sugar utilized by *Z. mobilis* ZMT2 in this study, pentoses such as xylose failed to be consumed during continuous enzymatic hydrolysis during ethanol fermentation, leading to a theoretical decrease in ethanol yield [21].

### Changes of the nitrogen source during different stages of corn straw cellulosic ethanol production

**Fig. 4:**
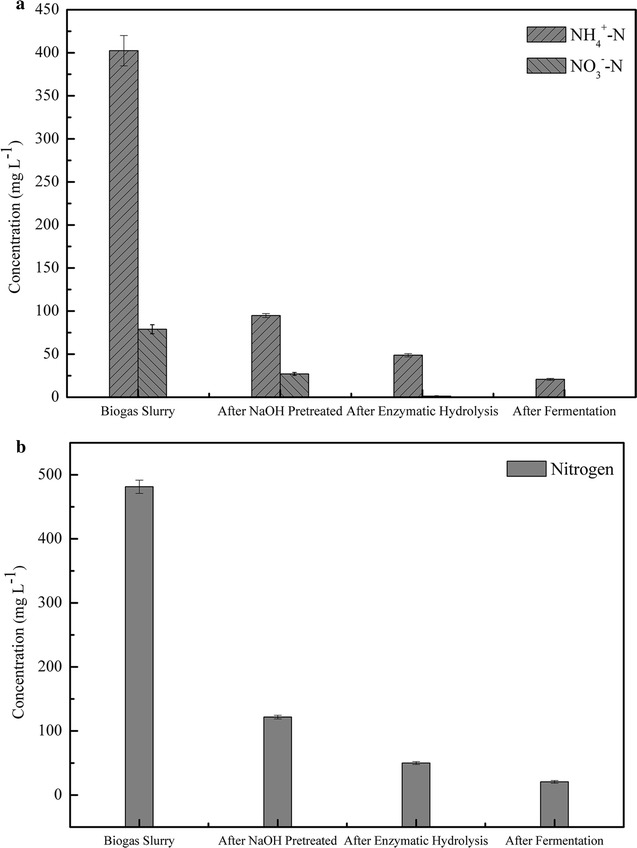
Ammonia nitrogen, nitrate nitrogen, and nitrogen content in the pretreated, enzymatically hydrolyzed, and fermented solutions. **a** Ammonia nitrogen and nitrate nitrogen content. **b** Nitrogen content. All data are the average of triplicates

Microbial growth and metabolism require adequate sources of nitrogen. Biogas slurry contains a large amount of nitrogen, which may be used for cell growth and metabolism during ethanol fermentation. *Z. mobilis* ZMT2 was able to ferment corn straw hydrolysate to produce ethanol without supplementing with additional nitrogen sources, indicating the nitrogen content in the enzymatic hydrolysate was sufficient for *Z. mobilis* growth and metabolism. Specifically, the biogas slurry contained 402.39 mg/L ammonia nitrogen, 78.9 mg/L nitrate nitrogen, and 0.036 mg/L nitrite nitrogen (Fig. 4a). After pretreatment, enzymatic hydrolysis, and ethanol fermentation, nearly 94.87 and 100% of the ammonia and nitrate nitrogen had been depleted, respectively, indicating that most of the nitrogen sources had been consumed by *Z. mobilis* ZMT2. In addition, element analysis indicates that the N content decreased from 481.29 to 20.65 mg/L after the final fermentation (Fig. 4b). Furthermore, there are 5.3% of the total glucose from corn straw hydrolysate was not consumed (Fig. 3b); we also attempted another experiment by addition (NH_4_)_2_SO_4_ to determine whether it was limiting the efficiency of sugar utilization. The results showed that nearly 95.02% of the total glucose has been utilized. It showed a slightly increased when compared to 100% biogas slurry.

Based on these results, biogas slurry could be used to replace process water and nitrogen sources during cellulosic ethanol production, helping to decrease the overall cost of cellulosic ethanol. This study revealed a novel and environmentally friendly treatment technology that uses biogas slurry, which helps to improve the value of biogas slurry.

Other industrial nitrogen sources have been commonly used for the production of cellulosic ethanol, including corn steep liquor (CSL) and diammonium phosphate (DAP), which have been estimated to incur costs between $1.7 and 2.2 million/year per 200 million liters ethanol plant [6, 16]. As shown in Table 6, the cost of CSL ranged from 18.3 to 35.6 cents/gallon, while the cost of process water ranged from 3.2 to 16.5 gal/gal ethanol and 18.01–110.06 cents/gal ethanol. The total cost of CSL and process water ranged from 47.31 to 141.86 cents/gal ethanol, which, in minimum ethanol selling price (MESP), was 17.65–42.99%.

**Table 6:** Cost of corn steep liquor and process water for use in cellulosic ethanol production

	1^a^	2^a^	3^a^	3 h^a^	4^a^	4 h^a^	5 h^a^
Cost of corn steep liquor (cents/gal ethanol)	31.8	19.4	35.6	32.7	33.8	29.3	18.3
Process water (gal/gal ethanol)	16.5	10.1	8.2	7.5	3.2	2.7	5.1
Cost of process water (cents/gal ethanol)	110.06	67.37	54.69	50.03	21.34	18.01	34.02
Total cost of corn steep liquor and process water (cents/gal ethanol)	141.86	86.77	90.29	82.73	55.14	47.31	52.32
Minimum ethanol selling price (MESP) in dollars per gallon ($/gallon)	3.3	3.09	3.15	2.95	3.03	2.68	2.87
Total cost of corn steep liquor and process water in Minimum ethanol selling price (MESP) (%)	42.99	28.08	28.66	28.04	18.2	17.65	18.23

Data from Ref. [17]

Whole slurry coculture SSF (Simultaneous Saccharification and Fermentation); Separate xylose and glucose fermentation; 3, 3 h, 4, 4 h, 5 h, Separate solid and liquid processing dilution of solids with ethanol from xylose fermentation or water

^a^Different technologies used for converting cellulosic material into ethanol

Therefore, a sustainable alternative process and nitrogen source are desired. In our previous work, dairy manure was evaluated as a carbon and nitrogen source for ethanol production [20]. In this present study, biogas slurry was used not to replace process water, but also to provide adequate nitrogen for cellulosic ethanol production. This inexpensive nitrogen source may play an important role in reducing the cost of cellulosic ethanol. If we use biogas slurry to replace fully all nitrogen source and process water, the total cost of nitrogen source and process water may be very little (only transport costs to ethanol plant). However, in other conventional process of cellulosic ethanol production (as shown in Table 6), the total costs of nitrogen source and process water are 17.65–42.99% of MESP. Presumably, using biogas slurry to replace process water and nitrogen may decrease the cost of cellulosic ethanol by nearly 10.0–20.0%. Further research on life cycle assessment and comparison of this method to other conventional cellulosic ethanol production pathways are also necessary on a large-scale cellulosic ethanol plant.

Importantly, the economic benefits of obtaining water and nitrogen from biogas slurry could extend beyond the production of cellulosic ethanol to the production of other chemicals. Interestingly, a previous study indicated that *Z. mobilis* could use N_2_ as a nitrogen source via N_2_ fixation [22]. In our previous and current work, dairy manure [20] and biogas slurry were effectively used as sources of nitrogen during cellulosic ethanol production by *Z. mobilis*. These studies on N_2_ fixation and our study on utilization of ammonia nitrogen in manure or biogas slurry by *Z. mobilis* uncover the advantage for using these during ethanol production as less expensive nitrogen sources. These studies not only enrich our understanding of biological nitrogen fixation, but also provide a theoretical and technical basis for using N_2_ and other alternative nitrogen sources with *Z. mobilis*.

### Mass balance and energy balance of the conversion of corn straw into ethanol

**Fig. 5:**
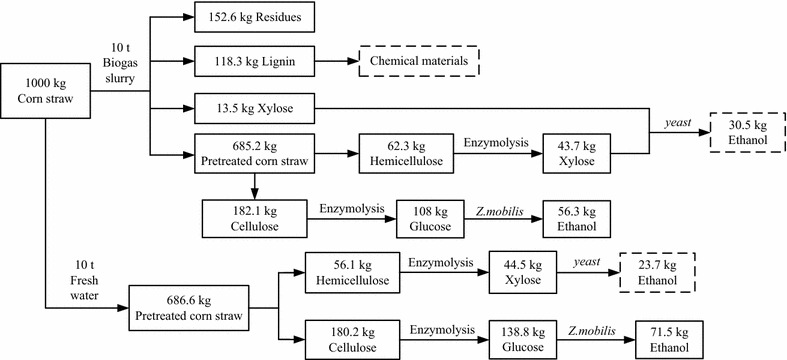
Laboratory scale mass balance of conversion of corn straw into ethanol

As shown in Fig. 5, pretreatment with 1.34% NaOH (w/v) diluted in 100% biogas slurry and the use of cellulase hydrolysis resulted in the production of 56.3 kg ethanol by *Z. mobilis* ZMT2 from 1000.0 kg of dried corn straw. In theory, the other 43.7 kg of xylose could be fermented by engineered yeast to produce 30.5 kg of ethanol [23]. When using pretreatment with 1.34% NaOH (w/v) diluted in water and cellulase hydrolysis, nearly 10.0 tons water and 18.0 kg yeast extract or other nitrogen source was required for 1000.0 kg corn straw. While 71.5 kg of ethanol could be produced by *Z. mobilis*, the remaining 44.5 kg of xylose could theoretically be fermented by engineered yeast to produce roughly equivalent yields of ethanol. Therefore, the process described in this work using biogas slurry during cellulosic ethanol production saved 10.0 tons process water and 18.0 kg nitrogen source. It means saved 210.0 yuan (RMB) when used biogas slurry for replacing process water and nitrogen source, and the whole net profit is 59.4 yuan. The main energy input and output in this study were also calculated [32]. As shown in Table 7, when biogas slurry used, the total energy input is 16.5 MJ/kg corn straw. However, in a conventional cellulosic ethanol production, the energy input is 17.29 MJ/kg corn straw. On the other hand, although the total energy output is only 2.58 MJ/kg corn straw when biogas slurry used. The net energy value is also higher than that of process water and nitrogen source application. With further optimization of the ethanol production process or consideration of other co-products production, higher net energy value can be achieved in the future. Taken together, using biogas slurry for replacing of process water and nitrogen source not only decreased the whole costs of cellulosic ethanol, but also increased the whole net energy value. In further studies, analysis of overall energy balance may also be necessary based on a large-scale cellulosic ethanol plant.

**Table 7:** Analysis of the main costs and energy balance of corn-to-ethanol in this study

	Fresh water			Biogas slurry		
	Amount	Costs (RMB)	Energy (kJ/kg)	Amount	Costs (RMB)	Energy (kJ/kg)
Main process inputs						
Corn straw	1000 kg	300^a^	16,350	1000 kg	300^a^	16,350
NaOH	134 kg	335^b^	148.91	134 kg	335^b^	148.91
Process water	10 m^3^	30^c^	42.0	0 m^3^	0	0
Biogas slurry	0 m^3^	0	0	10 m^3^	0	0
Nitrogen source	18 kg	180^d^	753.48	0 kg	0	0
Total costs (RMB)	− 845			− 635		
Total energy input (MJ/kg corn straw)			− 17.29			− 16.5
Main process outputs						
Ethanol	95.2 kg	761.6^e^		86.8 kg	694.4^e^	
Ethanol (Yuan/kg)		8.88			7.32	
Net profit (Yuan)		− 83.4			59.4	
Total energy output (MJ/kg corn straw)			2.83^f^			2.58^f^
Net energy value (MJ/kg corn straw)			− 14.46			− 13.92

^a, b, c, d^The costs of corn straw, NaOH, process water, biogas slurry, and nitrogen source are calculated based on the fact of China

^e^The output of ethanol is calculated as 8.0 Yuan/kg

^f^Energy value based on lower heating value

## Methods

### Materials and preparation

Corn straw was obtained from the Zheng Long farm in the Sichuan province and then stored at 4 °C in the lab until further use. Biogas slurry was obtained from a large-scale biogas plant in the Sichuan province and stored at 4 °C. Corn straw was collected, dried at 105 °C, pulverized into tiny particles, passed through a sieve with a 40-mesh screen, blended, and then packed. The amounts of neutral detergent fiber (NDF), acid detergent fiber (ADF), and acid detergent lignin (ADL) in the untreated and pretreated corn straw were determined using the National Renewable Energy Lab (NREL) method [24]. Changes in cellulose, hemicellulose, and ADL content are a reflection of NaOH pretreatment. The results from analysis of the untreated and pretreated corn straw are shown in Table 1.

### Pretreatment with NaOH diluted in biogas slurry

Corn straw was pretreated with biogas slurry mixed with NaOH solid. As shown in Table 2, the biogas slurry concentration was diluted with fresh water to 0–100% and then 0.67 g NaOH (1.34%, w/v) was added. In a hydrothermal reaction vessel, 5 g of untreated dried corn straw treated with 50 mL of different concentration of biogas slurry solution (ranging from 0 to 100%) with 1:10 solid:liquid ratio, as shown in Table 2. The samples were then incubated at 140 °C for 6 h. Each of the samples was tested at least three times in duplicate.

After pretreatment, the solid:liquid slurry was transferred into 50 ml centrifuge tubes, and then centrifuged at 4500 rpm/min for 3 min to separate the liquid and solid fractions. The lignocellulose content was calculated using the NREL method and the amount of reducing sugar in the supernatant was determined using the 3,5-dinitrosalicylic acid colorimetry method (DNS) [25]. Glucan and xylan contents were calculated based on the glucose and xylose concentrations using an hydro corrections of 0.9 [26]. The reducing sugar yield was calculated as follows:

$${\text{Yield of reducing sugar }}\left( \% \right) \, = \frac{{{\text{Reducing sugars released }} \times \, 0. 9}}{{{\text{Dry weight }} \times \, \left( {{\text{cellulose }} + {\text{ hemicellulose}}} \right)\% }} \times { 1}00\% .$$

### Bacterial strains and fermentation conditions

A stress tolerant strain, *Z. mobilis* ZMT2 [27] (CGMCC11888 from our lab, stored at the China General Microbiological Culture Collection Center), was cultured in rich media (RM) [28] at 30 °C without shaking. Cultures were maintained on glucose agar (20.0 g/L glucose, 10.0 g/L yeast extract, and 15.0 g/L agar). Organisms were subcultured in fresh inoculum media for 24 h at 30°C prior to inoculating into the fermentation medium. Inoculum medium (g/L) was comprised of yeast extract (10.0 g/L), MgCl_2_ (1.0 g/L), (NH_4_)_2_SO_4_ (1.0 g/L), KH_2_PO_4_ (1.0 g/L), and glucose (20.0 g/L).

### Tabletop scanning electron microscopy

During pretreatment, the structure of the corn straw was visualized by tabletop scanning electron microscopy using a TM-1000. Both the untreated straw and pretreated straw were dried at 105 °C for 5 h, mounted on conductive double-sided tape, and placed on the specimen stub of the microscope. An image was displayed following completion of the automatic function, where an accelerating voltage of 15 kV was used [29]. Structural changes were observed in untreated straw and pretreated straw.

### Enzymatic hydrolysis

Enzymatic hydrolysis was performed in 50 mL Erlenmeyer flasks containing 30 mL of a 50 mM sodium citrate buffer solution (pH 4.8) with 980 U/g enzyme and 10 g/L substrate. The flasks were incubated at 50 °C with shaking at 120 rpm for 72 h. A commercial enzyme solution (Sigma, CAS: 9012-54-8) from *Trichoderma reesei* ATCC 26921 containing 700 units/g was used for the enzymatic hydrolysis reaction.

### Continuous enzymatic hydrolysis

To enhance the sugar concentration for ethanol production, continuous enzymolysis was performed as described. Ten grams of corn straw pretreated with 100% biogas slurry was mixed with a suitable amount of cellulase, and then, 100 mL of sodium citrate buffer was added to the solution. Chloramphenicol was also added to prevent microbial contamination. Continuous enzymolysis was carried out for 144 h at 50 °C with shaking at 120 rpm in a 250 mL triangle bottle. The pretreated corn straw and cellulase were divided into three parts, and one part was added every 3 h. The sugar yield was measured by high-performance liquid chromatography (HPLC).

### Ethanol fermentation

*Zymomonas mobilis* ZMT2 was chosen as the ethanol producer by which to estimate fermentation of the continuous enzymatic hydrolysate [27]. Before inoculation, the continuous enzymatic hydrolysate was sterilized using aseptic membrane filtration. RM medium was used as a control against which to compare carbon and nitrogen use during ethanol fermentation. After inoculating to a final concentration of 10% inoculum, the fermentation experiment was performed at 30 °C without shaking.

### Analytical methods

The reducing sugar yield in the supernatant was analyzed by 2,4-dinitrosalicyclic acid (DNS) assay [25], while the monosaccharide composition was determined by comparing the retention time against standards using HPLC (Agilent), where an HPX-87H ion exclusion column (BioRad Aminex) was used at 35 °C with 5 mM H_2_SO_4_ as the mobile phase. A flow rate of 0.6 mL/min and an injection volume of 20 μL were used with a 30 min analysis time. HPLC was also used to analyze the glucose consumption and ethanol yield using the following equation [30]:

$${\text{Ethanol yield }}\left( \% \right) \, = \frac{{{\text{Final ethanol concentration }}\left( {{\text{g L}}^{ - 1} } \right)}}{{{\text{Initial glucose and xylose}} {\text{concentration}} \times 0. 5 1 1 { }\left( {{\text{g L}}^{ - 1} } \right)}} \times { 1}00\% .$$

The total nitrogen and carbon contents in pretreated, enzymatic hydrolyzed, and fermented solutions were analyzed by an spectrophotometry and auto analyzer (AA3, Bran + Luebbe, Norderstedt, Germany) [31].

All experimental data are presented as the mean of samples performed in triplicate, where error bars indicate standard deviation.

## Conclusions

In this study, biogas slurry was used to replace the process water and nitrogen sources required during cellulosic ethanol production. It was found that biogas slurry is an effective alternative to water during the corn straw preprocessing stage, and has important potential for applications in corn straw cellulosic ethanol production. This study not only provides a novel method of utilizing biogas slurry, but also a means by which to reduce the cost of cellulosic ethanol.

## Abbreviations

ED pathway: Entner–Doudoroff Pathway
CSL: corn steep liquor
DAP: diammonium phosphate
DNS: 3, 5-dinitrosalicylic acid colorimetry method
NDF: neutral detergent fiber
ADF: acid detergent fiber
ADL: acid detergent lignin
SSF: simultaneous saccharification and fermentation
HPLC: high-performance liquid chromatography
MESP: minimum ethanol selling price
